# PANSAID – PAracetamol and NSAID in combination: study protocol for a randomised trial

**DOI:** 10.1186/s13063-016-1749-7

**Published:** 2017-01-10

**Authors:** Kasper Højgaard Thybo, Daniel Hägi-Pedersen, Jørn Wetterslev, Jørgen Berg Dahl, Henrik Morville Schrøder, Hans Henrik Bülow, Jan Gottfrid Bjørck, Ole Mathiesen

**Affiliations:** 1 Department of Anaesthesiology, Næstved Hospital, Ringstedgade 61, 4700 Næstved, Denmark; 2 Copenhagen Trial Unit, Rigshospitalet, Department 7812, Blegdamsvej 9, 2100 Copenhagen Ø, Denmark; 3 Department of Anaesthesiology, Bispebjerg Hospital, Bispebjerg Bakke 23, 2400 Copenhagen, NV Denmark; 4 Department of Orthopedic Surgery, Næstved Hospital, Ringstedgade 61, 4700 Næstved, Denmark; 5 Department of Anaesthesiology, Holbæk Hospital, Smedelundsgade 60, 4300 Holbæk, Denmark; 6 Department of Orthopedic Surgery, Nykøbing Falster Hospital, Fjordvej 15, 4800 Nykøbing Falster, Denmark; 7 Department of Anaesthesiology, Zealand University Hospital Køge, Lykkebækvej 1, 4600 Køge, Denmark

**Keywords:** Ibuprofen, Paracetamol, Total hip arthroplasty, Benefit, Harm, Multimodal analgesia, Postoperative pain

## Abstract

**Background:**

Effective postoperative pain management is essential for the rehabilitation of the surgical patient. No ‘gold standard’ exists after total hip arthroplasty (THA) and combinations of different nonopioid medications are used with virtually no evidence for additional analgesic efficacy compared to monotherapy. The objective of this trial is to investigate the analgesic effects and safety of paracetamol and ibuprofen alone and in combination in different dosages after THA.

**Methods:**

PANSAID is a placebo-controlled, parallel four-group, multicentre trial with centralised computer-generated allocation sequence and allocation concealment and with varying block size and stratification by site. Blinding of assessor, investigator, caregivers, patients and statisticians. Patients are randomised to four groups: (A) paracetamol 1 g × 4 and ibuprofen 400 mg × 4, (B) paracetamol 1 g × 4 and placebo, (C) placebo and ibuprofen 400 mg × 4 and (D) paracetamol 0.5 g × 4 and ibuprofen 200 mg. The two co-primary outcomes are 24-h consumption of morphine and number of patients with one or more serious adverse events within 90 days after surgery. Secondary outcomes are pain scores during mobilisation and at rest at 6 and 24 h postoperatively, and number of patients with one or more adverse events within 24 h postoperatively. Inclusion criteria are patients scheduled for unilateral, primary THA; age above 18 years; ASA status 1–3; BMI >18 and <40 kg/m^2^; women must not be pregnant; and provision of informed consent. Exclusion criteria are patients who cannot cooperate with the trial; participation in another trial; patients who cannot understand/speak Danish; daily use of strong opioids; allergy against trial medication; contraindications against ibuprofen; alcohol and/or drug abuse. A total of 556 eligible patients are needed to detect a difference of 10 mg morphine i.v. the first 24 h postoperatively with a standard deviation of 20 mg and a family wise type 1 error rate of 0.025 (two-sided) and a type 2 error rate of 0.10 for the six possible comparisons of the four intervention groups.

**Discussion:**

We started recruiting patients in December 2015 and expect to finish in September 2017. Data analysis will be from September 2017 to October 2017 and manuscript submission ultimo 2017.

**Trial registration:**

EudraCT: 2015-002239-16 (12/8-15); ClinicalTrials.gov: NCT02571361. Registered on 7 October 2015.

**Electronic supplementary material:**

The online version of this article (doi:10.1186/s13063-016-1749-7) contains supplementary material, which is available to authorized users.

## Background

Effective postoperative pain management that promotes early mobilisation, fluid and food intake, and the resumptions of normal activities is essential for the wellbeing and rehabilitation of the surgical patient [1] and is a core component in enhanced recovery after surgery programmes [2, 3]. In daily clinical practice, patients are most often treated with different combinations of nonopioid drugs and analgesic methods (‘multimodal analgesia’) to achieve better analgesic effects and lower opioid requirements including their well-known adverse effects.

The medical literature on postoperative multimodal analgesia is, however, heterogenic and characterised by typically small studies using a variety of different combinations and techniques, and most often with short follow-up times that lower the probability of detecting relevant adverse effects. Consequently, most combinations of analgesics are not well-documented [4] and, therefore, it is a significant risk that patients’ pain is either treated insufficiently, or that patients receive combinations of analgesics without additive effects but with an increased risk of adverse effects [5]. It is documented that lack of systematic reporting of adverse events (AE) is frequent [5], yet, the rate of serious adverse events (SAEs) in a mixed orthopaedic population was found to be as high as 6.2% in a recent retrospective study [6].

The two most common drugs used as basic nonopioid analgesics after surgery are paracetamol and nonsteroidal anti-inflammatory drugs (NSAIDs). These drugs have a proven analgesic and morphine-sparing effect when administered individually [7, 8], whereas evidence of an additive and improved analgesic effect of their combination is virtually absent [4]. Furthermore, particularly the NSAIDs are associated with a number of potential adverse effects, e.g. gastrointestinal bleeding [9, 10], thromboembolic events [11, 12], impaired renal function [13], anastomotic leakage [14–17] and delayed bone-healing [18]. It has further been suggested that no safe treatment window exists, even for short periods of NSAID therapy, in patients with cardiovascular disease [19].

As stated above, the scientific evidence of a beneficial effect of the combined use of paracetamol and NSAIDs is limited. It is primarily based on two recent reviews: a systematic, qualitative review of 21 trials including a wide range of different pain models [20] and a Cochrane review with meta-analysis of three trials of dental surgical extraction [7] (Table 1).

**Table 1:** The most recent systematic reviews of combined paracetamol and a nonsteroidal anti-inflammatory drug (NSAID) versus paracetamol or a NSAID alone in postoperative pain management, NNT Number Needed to Treat

Study	Intervention	Number of patients	Surgery	Pain	Opioid	Adverse events
Derry 2013 [7]	Paracetamol and ibuprofen vs. ibuprofen	1647 (3 trials)	Extraction of at least 3 impacted third molars	Ibuprofen 200 mg and paracetamol 500 mg vs. placebo: NNT 1.6 (1.5–1.8) Ibuprofen 400 mg and paracetamol 1000 mg vs. placebo: NNT 1.5 (1.4–1.7) Ibuprofen 400 mg and paracetamol 1000 mg vs. ibuprofen 400 mg: NNT 5.4 (3.5–12.2)	Time to rescue medication: Ibuprofen 200 mg and paracetamol 500 mg: 7.6 h Ibuprofen 400 mg and paracetamol 1000 mg: 8.3 h Placebo: 1.7 h	No information
Ong 2010 [20]	Combinations of paracetamol and various NSAIDs vs. 1 of these drugs	1909 (21 trials)	Mixed surgical populations	Paracetamol and NSAID vs. paracetamol: 85% of studies showed that the combination had better analgesic properties than paracetamol alone Paracetamol and NSAID vs. NSAID: 64% of these studies showed that the combination had better analgesic properties than NSAID alone	Reduction in opioid consumption is not quantified in a combined measure	No systematic information

The review by Ong et al. [20] included 1909 patients and, based on the available data, it concludes that it was not possible to perform meta-analysis. They conclude that the combination of paracetamol and NSAIDs may provide superior analgesia compared to either drug alone. The review is limited by a qualitative approach including a wide range of acute pain models, and pooling of both minor and major surgical procedures in the analyses. Overall, 85% of trials comparing paracetamol and a NSAID versus paracetamol alone and 67% of trials comparing paracetamol and a NSAID versus a NSAID alone, provided more effective pain relief of the combination compared to single drugs.

The Cochrane review with meta-analysis [7] included 1647 patients, and investigated the combined effects of paracetamol and NSAIDs on established pain after dental surgery, with surgical removal of at least three impacted third molars. It was concluded that combination therapy (both ibuprofen 200 mg/paracetamol 500 mg and ibuprofen 400 mg/paracetamol 1000 mg) is more effective than placebo (Number Needed to Treat ((NNT) 1.6 and NNT 1.5, respectively) and that ibuprofen 400 mg/paracetamol 1000 mg is more effective than ibuprofen alone (NNT 5.4). No comparison of combination therapy versus paracetamol alone was included in this meta-analysis. The trials included only investigated relatively healthy and young adults.

Total hip arthroplasty (THA) is a common procedure and may be associated with moderate to intense postoperative pain. A large number of different treatment options have been investigated for pain following THA [21, 22] including opioids, corticosteroids, NSAIDs, paracetamol and gabapentinoids and various neuroaxial and peripheral nerve blocks. However, no established ‘gold standard’ for pain management after THA can be presented [21]. The most recent recommendation from postoppain.org includes paracetamol, NSAIDs (either COX-2 selective or mixed type) and opioids as rescue [23]. This trial will potentially provide valuable information about the optimal basic nonopioid combination regimen for pain management after THA.

In the present trial, we have chosen THA as the analgesic model for investigating benefit and harm of short-term treatment of paracetamol and NSAIDs in the trial: PANSAID – PAracetamol and NSAID in combination: a randomised, blinded, parallel four-group clinical trial.

### Aims

The aim of the PANSAID trial, is to investigate the analgesic effects and safety of paracetamol and ibuprofen and their combination in different dosages after THA.

## Methods/design

**Fig. 1:**
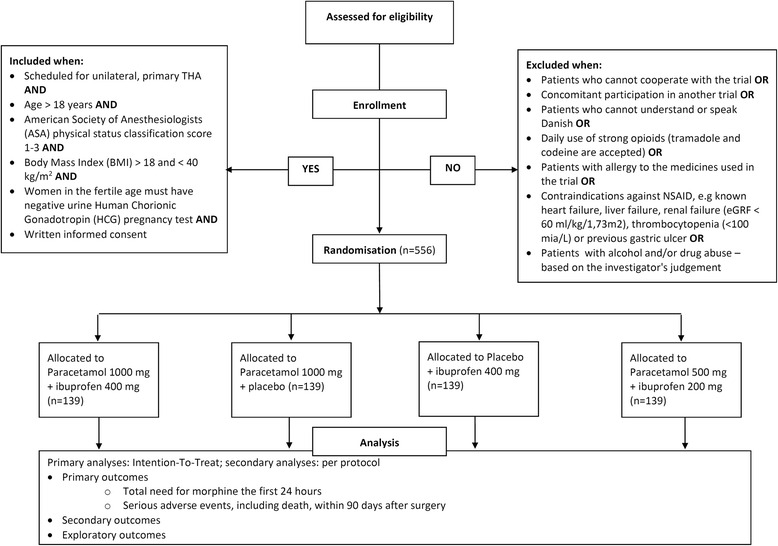
PANSAID flowchart

PANSAID is a randomised multicentre trial with a central computer-generated allocation sequence, concealed allocation, blinding of assessors, investigators, caregivers, patients and statisticians in patients having an elective total hip arthroplasty (Fig. 1). Patients will be randomised with varying block size and stratified according to site for treatment groups receiving either paracetamol and ibuprofen, paracetamol and placebo, placebo and ibuprofen or paracetamol and ibuprofen in a reduced dosage.

### Hypothesis

We hypothesise that the combination of paracetamol and ibuprofen is more effective than each drug alone and will reduce opioid consumption and/or pain levels. In addition, we hypothesise that a combination of lower doses of paracetamol and ibuprofen will demonstrate analgesic efficacy comparable to higher doses of each drug alone, and will reduce the risk of adverse effects per se.

### Analgesic interventions

NSAIDs have anti-inflammatory, antipyretic and analgesic properties and act by a reversible inhibition of the cyclooxygenase (COX) enzyme, which mediates the synthesis of prostaglandins and thromboxane A2. COX exists (primarily) in two isoforms, COX-1 and COX-2, and nonselective NSAIDs inhibit both isoforms (in varying degrees), while COX-2-selective NSAIDs inhibit mostly COX-2 [24, 25]. There are a number of potential adverse effects of NSAID treatment, including increased risk of cardiovascular events [11], gastrointestinal bleeding [9], ulcers [10], renal impairment [26], anastomotic leakage [14–17] and possible impaired bone-healing [18]. Furthermore, although the cardiovascular and gastrointestinal adverse effects are not well-characterised, there is some evidence that none of the NSAIDs are completely safe, especially for longer-term use [11, 27]. In the existing medical literature, most of these adverse effects are limited in description for short-term use in the perioperative setting, and a number of them may be related to NSAIDs with high COX-2 selectivity, e.g. diclofenac. The European Medicines Agency has recognised an increased cardiovascular risk in treatment with high-dose ibuprofen (more than 2400 mg per day) [28]. For postoperative pain treatment; it may, therefore, be safest to choose nonselective NSAIDs, like ibuprofen and naproxen, and in lowest effective dose and for as short a duration as possible, when NSAID treatment is needed.

Paracetamol has antipyretic and analgesic effects and is generally recommended as the first-line drug in nonopioid analgesic therapies including postoperative pain management. Its mode of action is still controversial and not yet fully understood. There may be a central effect via cannabinoid and vanilloid receptors and/or descending serotonergic pathways and, furthermore, a peripheral and central COX-inhibition has been proposed [29]. Although paracetamol is considered to have few adverse effects within the recommended dose range, liver failure is a known adverse effect in the setting of overdose or in specific patient populations (malnutrition, excess alcohol intake, etc.) [29].

Paracetamol and ibuprofen are available as ‘over-the-counter’ medications in many countries and are standard analgesic drugs used for postoperative pain treatment. In this trial, paracetamol is administered in total doses of 2000 mg and 4000 mg, and ibuprofen in doses of 800 mg and 1600 mg per day. For both paracetamol and ibuprofen these doses are within the range of normally recommended and used postoperative doses and regimens.

### Inclusion criteria

Patients must meet all the following criteria to be suitable for inclusion in the trial:

- Scheduled for unilateral, primary THA
- Aged over 18 years
- Have an American Society of Anesthesiologists (ASA) physical status classification score 1–3.
- Have a Body Mass Index (BMI) >18 and <40 kg/m^2^
- Women in the fertile age must have a negative urinary human chorionic gonadotropin (HCG) pregnancy test
- Patients must give written informed consent for participation in the trial after having fully understood the contents of the protocol and any restrictions

### Exclusion criteria

Patients who meet one or more of the following criteria are not suitable for inclusion in this trial:

- Patients who cannot cooperate with the trial (e.g. use of the PCA-pump, understand the Visual Analogue Scale (VAS) ruler, etc.)
- Concomitant participation in another trial
- Patients who cannot understand or speak Danish
- Daily use of strong opioids (tramadol and codeine are accepted)
- Patients with allergy to the medicines used in the trial
- Contraindications against NSAIDs and paracetamol, e.g. known heart failure, liver failure, renal failure (estimated glomerular filtration rate (eGRF) <60 ml/kg/1.73 m^2^), thrombocytopenia (<100 mia/L) or previous gastric ulcer
- Patients with alcohol and/or drug abuse – based on the investigator’s judgement

### Randomisation

Patients will be randomised to four groups at a 1:1:1:1 ratio with block randomisation of varying size and stratified by site. Copenhagen Trial Unit (CTU), Rigshospitalet, Denmark, provides a website for central computer randomisation. Each patient entering the trial will be given a unique randomisation number and a corresponding ‘trial medicine number’.

### Outcome measures

#### Co-primary outcomes

- Total need for morphine the first 24 h postoperatively administered as *both* patient-controlled analgesia (PCA) and supplemental morphine administered at the postanaesthesia unit the first hour postoperatively
- Serious adverse events, including death, within 90 days after surgery defined as a SAE (according to International Conference on Harmonisation-Good Clinical Practice (ICH-GCP) guidelines) except ‘prolongation of hospitalisation’

#### Secondary outcomes

- Pain scores (VAS) with active 30° flexion of the hip at 6 and 24 h postoperatively
- Pain scores at rest (VAS) at 6 and 24 h postoperatively
- Number of patients with one or more AE in the intervention period (0–24 h)

#### Exploratory outcomes

- Level of nausea at 6 and 24 h postoperatively
- Number of vomiting episodes (0–24 h) measured in the periods 0–6 and 6–24 h postoperatively
- Consumption of ondansetron in the period 0–24 h postoperatively
- Level of sedation at 6 and 24 h postoperatively
- Level of dizziness at 6 and 24 h postoperatively
- Blood loss during the surgical procedure (intraoperatively)
- Days alive and outside hospital within 90 days after surgery

#### Methods of measurements

The total dose of morphine (mg) in the period 0–24 h postoperatively, including PCA-morphine and nurse-administered supplemental morphine (bolus 2 mg) on patient request for the first postoperative hour, is recorded. Patients’ pain is recorded on a VAS of 100 mm, where 0 = no pain and 100 = worst possible pain. Pain is recorded at rest, and during 30° active flexion of the hip and at rest.

Nausea, sedation and dizziness are recorded on a verbal scale (none, mild, moderate, severe). The number of productive vomiting events (volume estimated over 10 ml) is recorded corresponding to the periods 0–6 and 6–24 h postoperatively by interview with the patient. Total use of ondansetron (mg) 0–24 h postoperatively is recorded. Patient-reported adverse effects are recorded, including gastrointestinal disturbances, neurological disturbances and elevated serum creatinine.

Ninety-day mortality rate is recorded from the civil registration system through Statistics Denmark. Serious adverse events (SAEs) are recorded from the Danish National Patient Registry. SAEs are defined as modified SAEs according to the ICH-GCP guidelines excluding ‘prolongation of hospitalisation’, as we recognise that it will be impossible to adjudicate such events.

Analgesic medication (paracetamol and NSAIDs) and need for medical attention from discharge to the end of trial period (90 days) are recorded from the patient questionnaire. At sites where a 3-month clinical control visit is part of the routine follow-up after THA the questionnaire is returned. If no such visit is routine the investigator at that site will telephone the patient or contact the patient by mail.

### Trial intervention

The trial period is from randomisation to 90 days postoperative. The intervention period is from randomisation to 24 h postoperative.

#### Treatment A

Paracetamol 1000 mg + ibuprofen 400 mg given per os starting 1 h before surgery and administered every 6 h postoperatively (±1 h), i.e. a total of four times the first postoperative day.

#### Treatment B

Paracetamol 1000 mg + placebo given per os starting 1 h before surgery and administered every 6 h postoperatively (±1 h), i.e. a total of four times the first postoperative day.

#### Treatment C

Placebo + ibuprofen 400 mg given per os starting 1 h before surgery and administered every 6 h postoperatively (±1 h), i.e. a total of four times the first postoperative day.

#### Treatment D

Paracetamol 500 mg + ibuprofen 200 mg given per os starting 1 h before surgery and administered every 6 h postoperatively (±1 h), i.e. a total of four times the first postoperative day.

### Concomitant medication/treatment

Standard premedication:

- None

Standard anaesthesia:

- For spinal anaesthesia bupivacaine 0.5% plain, 10–15 mg is used and no opioids are added. If sedation is needed propofol infusion is used

For general anaesthesia propofol infusion and remifentanil infusion are used as needed. If needed, sevoflurane-based anaesthesia will alternatively be allowed and recorded.

- Fifteen minutes before end of surgery sufentanil 0.3 mcg/kg intravenously (i.v.) is given to patients under general anaesthesia

Standard postoperative pain and nausea management:

- PCA-morphine, bolus 2 mg, lockout time 10 min. Mixture: morphine 1 mg/ml.
- If there is a need for morphine in addition to the PCA pump in the first 1 h postoperatively at the postanaesthesia care unit, additional bolus doses of 2 mg morphine i.v. can be given on request by the patient
- Ondansetron 4 mg i.v. is administered at the first indication of moderate-severe nausea and may then be supplemented with 1 mg i.v. Maximal total dose allowed is 8 mg over the first postoperative day

Pain treatment at the end of the intervention period will follow departmental guidelines.

Analgesic medications other than the PCA-morphine, including other opioids, chlorzoxazone, antidepressants, steroids and gabapentinoids (gabapentin or pregabalin), are not permitted during the intervention period. Gabapentinoids and antidepressants are only permitted if the patient continues an already instituted treatment from before surgery.

All nonanalgesic medications are permitted at the discretion of the attending physician.

### Blinding

The study medication will be masked by the pharmacy. Participants, those administrating the intervention, other caregivers, outcome assessors, data managers, statisticians and investigators drawing conclusions will be blinded to the intervention. The experimental medicine will be packed and labelled by the Capital Region Pharmacy in accordance with Good Manufacturing Practice (GMP) hereof. The trial medication is packed in one box per participant containing all medication for the intervention period. CTU retains the nonblinded list of the allocation sequence list stratified for sites, which will only be revealed for the investigators when the data has been analysed and abstracts [30] and conclusions covering the different possibilities for interpreting the trial results have been agreed upon by the Steering Committee/investigators. The investigators, as well as Jørn Wetterslev from CTU, have no access to the randomisation list.

### Safety

Adverse events (AE), adverse reactions (AR), serious adverse events (SAEs), serious adverse reactions (SARs) and suspected unexpected serious adverse reactions (SUSARs) will be recorded in the intervention period and will be reported to the relevant authorities according to guidelines from ICH-GCP and the Danish Medicines Agency.

### Participant withdrawal

#### Discontinuation of individual participants

If a SAE (according to the IHC-GCP definition) occurs in the intervention period (0–24 h postoperatively) and the investigator, after consultation with either principal investigator or sponsor, finds it infeasible for the patient to continue the trial, the medication will be discontinued and the participant will be asked whether we may still record data including follow-up data.

The blinding may only be broken if the continued treatment of the patient requires knowledge of the randomisation code. This can be done by the investigator without restrictions. Breaking of the code is done by contacting CTU by telephone.

#### Participant withdrawal

A patient who has not completed the trial is a patient included in the trial, i.e. one who has given informed consent, been randomised but withdraws the consent *and* does not allow for continued data recording after discontinuing the trial medications.

If a patient does not complete the trial an account is given as to whether and how this participant is followed in the trial – this also applies to dropouts – as well as what data has been collected from these participants. The patient will be asked if the withdrawal is only for the intervention/treatment and if they allow for further data registration or if withdrawal is also for any further data registration.

### Statistics

#### Sample size estimation

Due to six possible comparisons and a wish to limit the maximal family wise error rate to 0.025 (two-sided) for each of the co-primary outcomes, for a power of 90% we will need to randomise 556 patients (139 in each intervention group) to detect or to discard a minimal clinically relevant difference (10 mg) in 24-h morphine consumption with a standard deviation of 20 mg over 24 h. However, for the coprimary outcome of patients with one or more SAE we will collate events pending the use of ibuprofen or not corresponding to one comparison of 417 versus 139 patients. This comparison addresses possible harm due to the use of ibuprofen versus no use; a two-sided maximal type 1 error rate of 0.025 rendering a power of 80% to detect or discard an increase in the number of patients with one or more SAE from 10% to 20% will be used.

#### Statistical methods

The trial will be completed when 556 patients are included in the trial. The primary analysis will be a modified (excluding patients randomised but not operated) intention-to-treat comparing the co-primary outcome of opioid consumption between the four groups and the co-primary outcome of patients with one or more SAE between collated groups randomised to ibuprofen versus the group not receiving ibuprofen. If there are more than 5% missing data or patients lost to follow-up and Little’s test is statistically significant we will use multiple imputations (MI) to impute missing data [31]. Complete case analysis will be performed as well but the results of the analyses using MI-imputed datasets will be considered the primary result of the trial. The primary analysis of the continuous outcome of morphine consumption within 24 h will be nonparametric pair-wise comparisons between the median consumption of morphine between the four groups (six analyses) stratified for sites with the van Elteren test [32]. If possible 99.6% and 95% confidence intervals for the difference in medians will be provided by boot-strapping.

Per-protocol analyses excluding patients with major protocol violations will also be performed. Major protocol violations are defined below. Per-protocol analyses regarding SAEs and other safety variables will, however, include patients with major protocol violation definition number 3 (below).

Major protocol violations will be defined as:

1. Patients who did not get any of the dosages of the randomised allocated trial treatment
2. Patients withdrawing from the trial intervention but allowing the use of registered data
3. Patients undergoing surgery (besides the elective THA) *or* a procedure in the intervention period that requires anaesthesia or sedation and/or analgesia

The evaluability assessment of each patient in the statistical analyses will be performed before the code is broken. Excluded patients and missing, unused or false data will be described. Data will be stored and evaluation and statistical analysis will be made by a statistician blinded for the interventions, where patient anonymity will be preserved and local data legislation will be observed.

Any change to the statistical plan will be accounted for by publication. A detailed statistical analysis plan will be published.

### Data collection

**Fig. 2:**
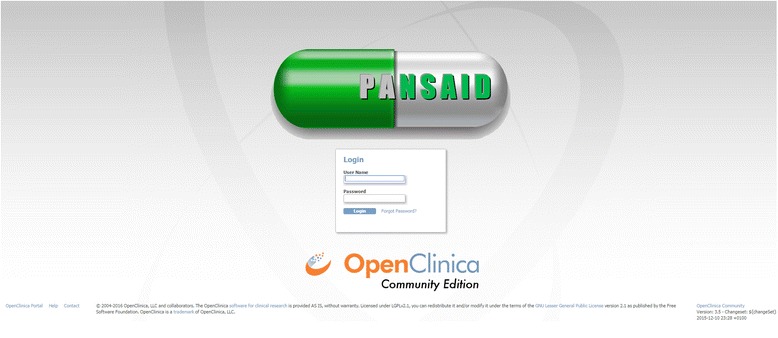
Screenshot of the electronic Case Report Form (eCRF)

All data will be entered into an electronic Case Report Form (eCRF) created and maintained by CTU (Fig. 2). The eCRF will form the basis for the electronic database. Data will be collected directly from the patients by trial investigators or clinical personnel educated and monitored by trial investigators and from the electronic anaesthesia chart, the electronic patients chart, the civil registration system through Statistics Denmark, and the Danish National Patient Registry. All data will be handled according to The Danish Data Protection agency and local original data (e.g. Informed Consent Forms) and records will be stored at trials sites for 5 years after the completion of the study. The study database will be anonymised and made publically accessible 18 months following publication of the study.

### Monitoring

The trial will be externally monitored by the University of Copenhagen Good Clinical Practice (GCP) Unit according to the latest legislation.

### Ethical consideration

The trial will be conducted in accordance with the principles of the Declaration of Helsinki and in compliance with the protocol approved by the competent authority and Ethics Committee, and according to GCP standards [33]. No deviation from the protocol will be implemented without the prior review and approval of the regulatory authorities except where it may be necessary to eliminate an immediate hazard to the trial participants.

### Data analysis and publication

Prior to breaking of the randomisation code an independent statistician will perform the data analysis according to a detailed statistical analysis plan. Based on the masked result the Steering Committee will agree upon abstracts covering all possible combinations and then the blinding will be broken. The final manuscript will contain the correct pre-made abstract. The protocol followed the Standard Protocol Items: Recommendations for Interventional trials (Additional file 1) [34] and the manuscript will follow Consolidated Standards Of reporting of Randomised Trials (CONSORT Statement) [35]. Authorship will be granted following the guidelines from the International Committee of Medical Journal Editors (ICMJE) [36]. Funding sources will have no influences on the interpretation of data.

The full, anonymised dataset will be published no longer than 18 months after completion of the trial.

The trial is registered at clinicaltrials.gov with identifier: NCT02571361.

### Substudies

We preplan the following substudies:

1. A reanalysis of benefit outcomes (pain and opioid consumption) with respect to the following subgroups: sex, age, ASA-score, type of surgery (uncemented, cemented or hybrid), surgical site (posterior approach versus anterolateral approach) and anaesthetic technique (general anaesthesia versus spinal anaesthesia)
2. A reanalysis of harm (AEs and opioid-related side effects) with respect to the following groups sex, age, ASA-score, anaesthetic technique (general anaesthesia versus spinal anaesthesia) and opioid consumption (in the intervention period)
3. Longer follow-up than the specified 90 days (1 year)
4. An analysis of the association between VAS scores and opioid consumption
5. Time-to-event analyses regarding use of PCA-morphine
6. An analysis of the association between preoperative analgesic use and pain/morphine consumption
7. An analysis of the individual patients: how many will achieve ‘no worse than mild pain’ (NRS <3)

More substudies may be performed post hoc and they will be clearly identified as such.

### Timeline

2015: application for approval from the Danish Medicines Agency, the Ethics Committee and the Danish Data Registration Agency. Development of an eCRF and randomisation website

2015–2017: inclusion of patients

2018: data analysis, writing and submission of the manuscript

## Discussion

PANSAID will provide the first, large, high-quality data regarding the combination of paracetamol and ibuprofen used in a surgical setting. We expect this trial to supply a significant contribution to a systematic and evidence-based approach towards nonopioid multimodal analgesic regimens for postsurgical treatment in a broader context.

## Trial status

**Fig. 3:**
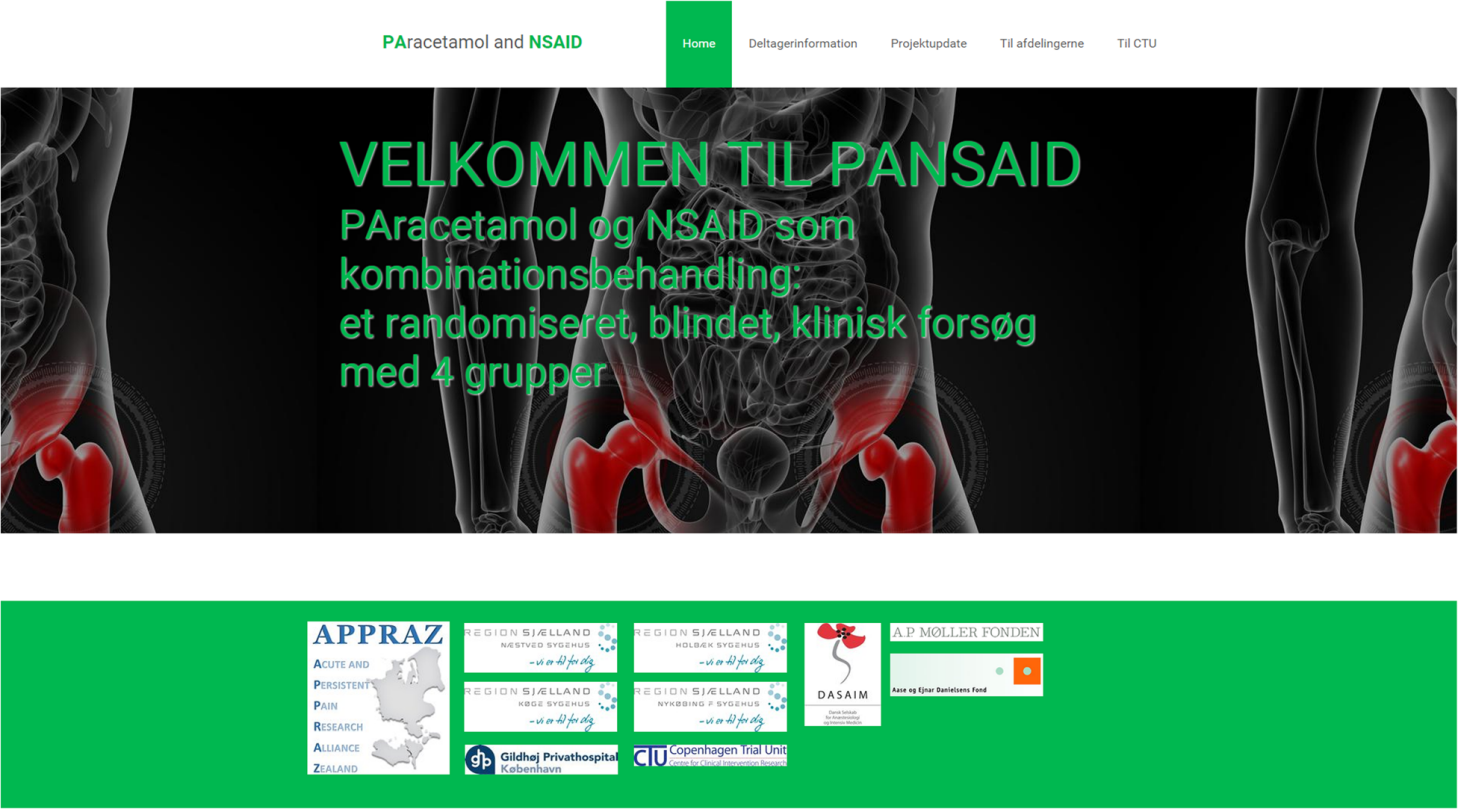
Screenshot of the trial homepage, www.pansaid.dk

Currently, more than 200 patients have been enrolled in the trial. The trial status can be seen at the trial website www.pansaid.dk (Fig. 3). We expect the enrolment period to end in September 2017.

## Additional file

Additional file 1: SPIRIT 2013 Checklist: Recommended items to address in a clinical trial protocol and related documents*. SPIRIT figure: PANSAID. (ZIP 47 kb)

## Abbreviations

AE: Adverse events
AR: Adverse reactions
ASA: American Society of Anesthesiologists
BMI: Body Mass Index
CONSORT: Consolidated Standards of Reporting Trials
COX: Cyclooxygenase
CTU: Copenhagen Trial Unit
DASAIM: The Danish Society of Anaesthesiology and Intensive care Medicine
eCRF: Electronic Case Report Form
eGRF: Estimated glomerular filtration rate
GCP: Good Clinical Practice
GMP: Good Manufacturing Practice
HCG: Human chorionic gonadotropin
ICH: The International Conference on Harmonisation of technical requirements for registration of pharmaceuticals for human use
ICMJE: International Committee of Medical Journal Editors
MI: Multiple imputations
NSAID: Nonsteroidal anti-inflammatory drugs
PCA: Patient-controlled analgesia
SAE: Serious adverse event
SAR: Serious adverse reaction
SPIRIT: Standard Protocol Items: Recommendations for Interventional Trials
SUSAR: Suspected unexpected serious adverse reaction
THA: Total hip arthroplasty
VAS: Visual Analogue Scale
