# Supplementary material for: PANSAID – PAracetamol and NSAID in combination: study protocol for a randomised trial
Source: Trials. 2017 Jan 10;18:11. doi: 10.1186/s13063-016-1749-7 (PMC5223299; doi:10.1186/s13063-016-1749-7)
Supplement: Additional file 1: — SPIRIT 2013 Checklist: Recommended items to address in a clinical trial protocol and related documents*. SPIRIT figure: PANSAID. (ZIP 47 kb) [file 13063_2016_1749_MOESM1_ESM.zip › Additional file 1/SPIRIT-Figure PANSAIDv2.doc]

SPIRIT figure: PANSAID

|  | Study period | | | | | | |
| --- | --- | --- | --- | --- | --- | --- | --- |
|  | | Enrolment | Allocation | Post-allocation | | | |
| Timepoint | | 2 months to 0 months pre surgery | T=1hour pre surgery | T=0, end of surgery | T=6 hours post surgery | T=24 hours post surgery | T=90 days post surgery |
| Enrolment: | |  |  |  |  |  |  |
| Eligibility screen | | x |  |  |  |  |  |
| Informed consent | | x |  |  |  |  |  |
| Allocation | |  | X |  |  |  |  |
| Intervention: | |  |  |  |  |  |  |
| Treatment A | |  |  |  |  |  |  |
| Treatment B | |  |  |  |  |  |  |
| Treatment C | |  |  |  |  |  |  |
| Treatment D | |  |  |  |  |  |  |
| Assessments: | |  |  |  |  |  |  |
| Efficacy variables | |  |  |  |  |  |  |
| Safety variables | |  |  |  |  |  |  |
